# Characteristics of critically ill patients with COVID-19 in the intensive care unit of a peruvian hospital

**DOI:** 10.17843/rpmesp.2022.391.10279

**Published:** 2022-03-31

**Authors:** Abel Arroyo-Sánchez

**Affiliations:** 1 Hospital Víctor Lazarte Echegaray, Seguro Social de Salud, Trujillo, Peru. Hospital Víctor Lazarte Echegaray Seguro Social de Salud Trujillo Peru; 2 Escuela de Medicina Humana, Universidad Privada Antenor Orrego, Trujillo, Peru. Universidad Privada Antenor Orrego Escuela de Medicina Humana Universidad Privada Antenor Orrego Trujillo Peru

*To the editor*. Severe coronavirus disease 2019 (COVID-19) is associated with acute respiratory distress syndrome (ARDS), which may require the use of invasive mechanical ventilation (IMV) ^1^. The Victor Lazarte Echegaray Hospital is a general tertiary care center where a ten-bed intensive care unit (ICU) was created to care for critically ill patients with COVID-19 and moderate or severe ARDS ^1^ requiring IMV. Treatment was framed within the recommendations of the World Health Organization ^1^. The aim of this letter was to describe the characteristics, therapeutic measures and outcomes of critically ill patients with COVID-19 on IMV and to compare them with similar studies.

We carried out a descriptive study on patients who attended the hospital from April 11 to June 30, 2021 and followed up until discharge. The medical records of patients aged 18 years and older were included and patients who died within 48 hours of admission were excluded. Frequencies and percentages were estimated for categorical variables and mean (standard deviation) or median (interquartile range 25-75) for quantitative variables. We compared variables with normal and non-normal distribution by using Student’s t-test and Mann Whitney U-test, respectively. SPSS version 25 was used. This study was approved by the Institutional Ethics Committee.

Thirty-five patients were included, 22.9% were 60 years of age or older; no patient died within the first 48 hours. Demographic, clinical and laboratory characteristics according to hospital discharge status are shown in Table 1. Availability of laboratory tests allowed measurement of ferritin concentration in 21 patients, fibrinogen in 17 patients, lactate dehydrogenase in 27 patients, C-reactive protein in 28 patients, and albumin in 30 patients. Computed tomography of the lungs was performed in 33 patients and the mean percentage of pulmonary involvement was 64.1% (SD: 14.2; range 35 - 95%).

**Table 1:** Demographic, clinical, laboratory, therapeutic characteristics, as well as severity and length of stay according to hospital discharge status of critically ill patients with critical COVID-19.

Variable	Status at hospital discharge		Total n=35
	Survivors n=27	Deceased n=8	
Age (years) ^a^	46.9 (12.5)	56.4 (12.7)	49.1 (12.9)
Sex, n (%)			
Male	18 (66.7)	7 (87.5)	25 (71.4)
Female	9 (33.3)	1 (12.5)	10 (28.6)
Confirmatory laboratory test, n (%)			
Antigenic	25 (92.6)	6 (75.0)	31 (88.6)
Molecular	2 (7.4)	2 (25.0)	4 (11.4)
Days of illness prior to admission ^a^	14.0 (4.9)	14.8 (2.9)	14.2 (4.5)
Comorbidities, n (%)			
Arterial hypertension	6 (22.2)	2 (25.0)	8 (22.9)
Diabetes mellitus	5 (18.5)	3 (37.5)	8 (22.9)
Immunosuppression	1 (3.7)	0 (0.0)	1 (2.9)
Obesity	15 (55.6)	3 (37.5)	18 (51.4)
Other	8 (29.6)	5 (62.5)	13 (37.1)
None	6 (22.2)	1 (12.5)	7 (20.0)
Laboratory			
O positive blood type, n (%)	12 (44.3)	5 (62.5)	17 (48.6)
Leucocytes (x103)/Μl ^a^	12.89 (5.88)	16.63 (8.89)	13.75 (6.72)
Lymphocytes (x103)/Μl ^b^	0.92 (0.63-1.43)	0.69 (0.32-1.20)	0.80 (0.63-1.47)
Platelets (x103)/Μl ^a^	345.9 (125.4)	265.1 (104.7)	327.5 (124.4)
Ferritin (ng/dL) ^a^	1261.0 (983.6)	1567.8 (845.8)	1334.0 (941.6)
Fibrinogen (mg/dL) ^b^	602.5 (556.3-685.5)	649.0 (565.5-689.0)	625.0 (562.5-698.0)
Lactate dehydrogenase (U/L) ^a^	739.2 (341.9)	984.9 (258.9)	802.9 (336.1)
C-reactive protein (mg/dL) ^b^	102.0 (42.3-202.3)	57.0 (37.0-145.8)	84 (40.8-191.5)
PaO_2_ / FiO_2_^b^	103 (86-139)	90 (58-134)	103 (82-139)
Creatinine (mg/dL) ^b^	0.7 (0.6-0.8)	0.8 (0.7-0.9)	0.7 (0.6-0.9)
Albumin (gr/dL) ^a^	3.33 (0.35)	2.98 (0.43) ^c^	3.26 (0.38)
Total bilirubin (mg/dL) ^a^	0.51 (0.18)	0.81 (0.19) ^d^	0.58 (0.22)
Severity scores			
APACHE II ^a^	13.2 (6.2)	16.8 (3.6)	14.0 (5.8)
SOFA ^b^	5.0 (4.0-7.0)	6.0 (4.0-8.0)	5.0 (4,0-7.0)
Tomographic lung involvement percentage ^a^	62.9 (13.4)	68.6 (17.3)	64.1 (14.2)
Glasgow Coma Scale ^b^	15.0 (15.0-15.0)	14.5 (13.3-15.0) ^e^	15.0 (15.0-15.0)
Days of treatment prior to admission			
Enoxaparin ^b^	3.0 (2.0-5.0)	3.0 (2.0-5.0)	4.0 (2.0-4.0)
Dexamethasone ^b^	5.5 (3.5-7.8)	5.5 (3.5-7.8)	4.0 (2.0-4.0)
Treatment at admission, n (%)			
Vasopressor	8 (29.6)	4 (50.5)	12 (34.3)
Antibiotic therapy	9 (33.3)	3 (37.5)	12 (34.3)
IMV in prone position	22 (81.5)	7 (87.5)	29 (82.9)
Days of stay			
Intensive care unit ^b^	17.0 (12.0-33.0)	18.0 (13.3-32.8)	17 (13-33)
Hospital ^b^	25.0 (22.0-41.0)	18.0 (14.0-36.8)	25 (20-40)

PaO_2_/FiO_2_: arterial oxygen pressure divided by inspiratory oxygen fraction; IMV: invasive mechanical ventilation.

a Mean (standard deviation)

b Median (interquartile range)

c Student’s t-test, p = 0.049

d Student’s t-test, p = 0.001

e Mann Whitney U Test, p = 0.001

Of the patients, 88.6% were admitted with history of having received dexamethasone and enoxaparin. All cases completed 10 days of treatment with dexamethasone and received thromboprophylaxis during the remainder of their stay. Due to a favorable oxygenation response with PaO_2_/FiO_2_ > 150 at the start of IMV, 6 patients underwent IMV in the supine position; the remaining 29 patients were placed in the prone position.

The in-hospital and ICU mortality rate was 22.9% (8 of 35 patients) and 37.5% in patients aged 60 years and older. We observed a significant association between lower albumin value (p = 0.049), lower Glasgow Coma Scale score (p = 0.001) and higher total bilirubin value (p < 0.001) in the deceased compared to the survivors (Table 1).

The mean age of the patients (49.1; SD: 12.9 years) and the prevalence of male sex (71.4%) were similar to those described in patients with COVID-19 undergoing IMV ^2^ ^-^ ^6^. The mean number of days of illness before ICU admission (14.2; SD: 4.5 days) was higher than what was described by Søvik *et al*. ^3^, Krause *et al*. ^4^ and Ñamendys-Silva *et al*. ^5^.

We found that the prevalence of obesity (51.4%) was higher than what was described in Norway (30.6%) ^3^, but lower than that reported in the United States (68%) ^4^. Arterial hypertension and diabetes mellitus had lower prevalence than what was reported by other investigators ^3^ ^-^ ^5^.

Among the clinical and laboratory findings of the organs evaluated with the SOFA score, the median PaO_2_/FiO_2_ (103; 86 - 139) was found to be lower than what was reported by Ziehr *et al*. ^2^ and Søvik *et al*. ^3^. The need for vasopressor on admission (34.3%) was lower than that reported by Ziehr *et al*. ^2^ and Ñamendys-Silva *et al*. ^5^. Plasma creatinine (0.7; 0.6 - 0.9 mg/dL) and total bilirubin (0.58; SD: 0.22 mg/dL) were lower, although the mean platelet count (327.5; SD: 124.4 x103/µL) was higher than that described by Ñamendys-Silva *et al*. ^5^.

Antibiotic use on admission was observed in 34.3% of patients which was lower than what was reported in the United States ^2^ and Mexico ^5^. Of the patients, 82.9% required IMV in prone position and the indication was PaO_2_/FiO_2_ ≤ 150 postintubation, the proportion of patients in prone IMV was higher than that described by other authors ^2^ ^-^ ^5^. The programming of IMV was made according to the predicted weight of the patient ^1^, in case of IMV in prone position, the session lasted 72 h.

Mortality (22.9%) was lower than what has been described in similar studies ^4^ ^-^ ^6^ and higher if compared to studies where mortality was assessed at 30 days ^2^ ^,^ ^3^, the aforementioned studies were conducted during the first wave of the pandemic and before the existence of the vaccine for COVID-19. One patient in our series had received the first dose of vaccine, had history of bone marrow transplant for multiple myeloma and died from COVID-19. The probable explanation for the lower mortality in this study may be the younger age of the patients, the experience acquired during the first wave, the lower frequency of diabetic and cardiac patients, non-abuse of antibiotics on admission, and IMV according to individual characteristics. None of the patients who died in the ICU could be removed from IMV. The median hospital stay (25; 17-36 days) and the median ICU stay (17; 13-33 days) were similar to what was found by Søvik *et al*. ^3^ and longer than that described by Ñamendys-Silva *et al*. ^5^.

Our study has the limitations inherent to the used methodology (observational and retrospective), the sample size, the fact that it was conducted in a single center, and the lack of diagnostic support tests in some patients.

In conclusion, our population was younger, received IMV in prone position more frequently and had a lower mortality rate, although with a longer hospital and ICU stay, when compared to other published reports.
