# Supplementary material for: Characteristics of critically ill patients with COVID-19 in the intensive care unit of a peruvian hospital
Source: Rev Peru Med Exp Salud Publica. 2022 Mar 31;39(1):117–9. doi: 10.17843/rpmesp.2022.391.10279 (PMC11397745; doi:10.17843/rpmesp.2022.391.10279)
Supplement: Translation

# Translation: Características de los pacientes críticos con COVID-19 en cuidados intensivos de un hospital peruano

**Authors:** Abel Arroyo-Sánchez

**Affiliations:** 1 Hospital Víctor Lazarte Echegaray, Seguro Social de Salud, Trujillo, Perú.; 2 Escuela de Medicina Humana, Universidad Privada Antenor Orrego, Trujillo, Perú.

Sr. Editor. La enfermedad por coronavirus 2019 (COVID-19) crítica, cursa con síndrome de dificultad respiratoria aguda (SDRA) que puede requerir el uso de ventilación mecánica invasiva (VMI) ^1^. El hospital Víctor Lazarte Echegaray es un centro para atención general de tercer nivel en donde se creó una unidad de cuidados intensivos (UCI) de diez camas para atender pacientes críticos con COVID-19 y SDRA moderado o grave ^1^ que requerían VMI. El tratamiento estuvo enmarcado en las recomendaciones de la Organización Mundial de Salud ^1^. El objetivo de esta carta es describir las características, las medidas terapéuticas y los resultados de los pacientes críticos con COVID-19 en VMI y compararlas con estudios similares.

Se realizó un estudio descriptivo en pacientes atendidos del 11 de abril al 30 de junio de 2021 y se hizo el seguimiento hasta el alta hospitalaria. Se incluyeron las historias clínicas de pacientes de 18 a más años y se excluyeron a pacientes fallecidos dentro de las 48 horas de admisión. Se estimaron frecuencias y porcentajes para las variables categóricas y la media (desviación estándar) o la mediana (rango intercuartílico 25-75) para las variables cuantitativas. La comparación de las variables con distribución normal y no normal se hicieron con las pruebas T de Student y U de Mann Whitney, respectivamente. Se usó el programa SPSS versión 25. El presente estudio fue aprobado por el Comité de Ética Institucional.

Se incluyeron 35 pacientes, el 22,9% tuvieron 60 a más años, ningún paciente falleció dentro de las primeras 48 horas Las características demográficas, clínicas y de laboratorio de acuerdo con la condición del alta hospitalaria se muestran en la Tabla 1. La disponibilidad de los exámenes de laboratorio permitió medir la concentración de ferritina en 21 pacientes, fibrinógeno en 17 pacientes, deshidrogenasa láctica en 27 pacientes, proteína C reactiva en 28 pacientes y la albúmina en 30 pacientes. La tomografía computarizada de pulmones se realizó en 33 pacientes y el porcentaje de compromiso pulmonar medio fue de 64,1% (DE: 14,2; rango 35 - 95%).

**Tabla 1:** Características demográficas, clínicas, laboratorio, severidad, terapéuticas y de estancia de acuerdo con la condición de alta hospitalaria de los pacientes críticos con COVID-19 crítico.

Variable	Condición de alta hospitalaria		Total n = 35
	Vivos n = 27	Fallecidos n = 8	
Edad (años) ^a^	46,9 (12,5)	56,4 (12,7)	49,1 (12,9)
Sexo, n (%)			
Masculino	18 (66,7)	7 (87,5)	25 (71,4)
Femenino	9 (33,3)	1 (12,5)	10 (28,6)
Prueba de laboratorio confirmatoria, n (%)			
Antigénica	25 (92,6)	6 (75,0)	31 (88,6)
Molecular	2 (7,4)	2 (25,0)	4 (11,4)
Días de enfermedad previos al ingreso ^a^	14,0 (4,9)	14,8 (2,9)	14,2 (4,5)
Comorbilidades, n (%)			
Hipertensión arterial	6 (22,2)	2 (25,0)	8 (22,9)
Diabetes mellitus	5 (18,5)	3 (37,5)	8 (22,9)
Inmunosupresión	1 (3,7)	0 (0,0)	1 (2,9)
Obesidad	15 (55,6)	3 (37,5)	18 (51,4)
Otra	8 (29,6)	5 (62,5)	13 (37,1)
Ninguna	6 (22,2)	1 (12,5)	7 (20,0)
Laboratorio			
Grupo sanguíneo O positivo, n (%)	12 (44,3)	5 (62,5)	17 (48,6)
Leucocitos (x10^3^)/μL ^a^	12,89 (5,88)	16,63 (8,89)	13,75 (6,72)
Linfocitos (x10^3^)/μL ^b^	0,92 (0,63-1,43)	0,69 (0,32-1,20)	0,80 (0,63-1,47)
Plaquetas (x10^3^)/μL ^a^	345,9 (125,4)	265,1 (104,7)	327,5 (124,4)
Ferritina (ng/dL) ^a^	1261,0 (983,6)	1567,8 (845,8)	1334,0 (941,6)
Fibrinógeno (mg/dL) ^b^	602,5 (556,3-685,5)	649,0 (565,5-689,0)	625,0 (562,5-698,0)
Deshidrogenasa láctica (U/L) ^a^	739,2 (341,9)	984,9 (258,9)	802,9 (336,1)
Proteína C reactiva (mg/dL) ^b^	102,0 (42,3-202,3)	57,0 (37,0-145,8)	84 (40,8-191,5)
PaO2 / FiO2 ^b^	103 (86-139)	90 (58-134)	103 (82-139)
Creatinina (mg/dL) ^b^	0,7 (0,6-0,8)	0,8 (0,7-0,9)	0,7 (0,6-0,9)
Albúmina (gr/dL) ^a^	3,33 (0,35)	2,98 (0,43)^ c^	3,26 (0,38)
Bilirrubina total (mg/dL) ^a^	0,51 (0,18)	0,81 (0,19)^ d^	0,58 (0,22)
Puntajes de severidad			
APACHE II ^a^	13,2 (6,2)	16,8 (3,6)	14,0 (5,8)
SOFA ^b^	5,0 (4,0-7,0)	6,0 (4,0-8,0)	5,0 (4,0-7,0)
% compromiso pulmonar tomográfico ^a^	62,9 (13,4)	68,6 (17,3)	64,1 (14,2)
Escala de coma de Glasgow ^b^	15,0 (15,0-15,0)	14,5 (13,3-15,0)^ e^	15,0 (15,0-15,0)
Días de tratamiento previo al ingreso			
Enoxaparina ^b^	3,0 (2,0-5,0)	3,0 (2,0-5,0)	4,0 (2,0-4,0)
Dexametasona ^b^	5,5 (3,5-7,8)	5,5 (3,5-7,8)	4,0 (2,0-4,0)
Tratamiento al ingresar, n (%)			
Vasopresor	8 (29,6)	4 (50,5)	12 (34,3)
Antibioticoterapia	9 (33,3)	3 (37,5)	12 (34,3)
VMI en posición prona	22 (81,5)	7 (87,5)	29 (82,9)
Días de estancia			
Unidad de cuidados intensivos ^b^	17,0 (12,0-33,0)	18,0 (13,3-32,8)	17 (13-33)
Hospitalarios ^b^	25,0 (22,0-41,0)	18,0 (14,0-36,8)	25 (20-40)

PaO_2_/FiO_2_: presión arterial de oxígeno entre la fracción inspiratoria de oxígeno; VMI: ventilación mecánica invasiva

a Media (desviación estándar)

b Mediana (rango intercuartílico)

c Prueba T de Student, p = 0,049

d Prueba T de Student, p = 0,001

e Prueba U de Mann Whitney, p = 0,001

El 88,6% de los pacientes ingresaron con el antecedente de haber recibido dexametasona y enoxaparina. En todos los casos se completaron los diez días de dexametasona y se brindó tromboprofilaxis durante el resto de su estancia. En seis pacientes se realizó VMI en posición decúbito supina, debido a una respuesta oxigenatoria favorable con PaO_2_/FiO_2_ > 150 al iniciar la VMI, los 29 pacientes restantes fueron colocados en posición decúbito prono.

La mortalidad intrahospitalaria y en la UCI fue de 22,9% (8 de 35 pacientes) y en los pacientes de 60 a más años fue del 37,5%. Se evidenció una asociación significativa entre el menor valor de albúmina (p = 0,049), el menor puntaje en la escala de coma de Glasgow (p = 0,001) y el mayor valor de bilirrubina total (p < 0,001) en los fallecidos en comparación con los vivos (Tabla 1).

La edad media de los pacientes (49,1; DE: 12,9 años) y la prevalencia del sexo masculino (71,4%) fueron semejantes a las descritas en pacientes con COVID-19 sometidos a VMI ^2^ ^-^ ^6^. La media de días de enfermedad antes del ingreso a la UCI (14,2; DE: 4,5 días) fue mayor a lo descrito por Søvik *et al.* ^3^, Krause *et al.* ^4^ y Ñamendys-Silva et al. ^5^.

La prevalencia de obesidad (51,4%) fue mayor a la descrita en Noruega (30,6%) ^3^, pero menor a la reportada en Estados Unidos (68%) ^4^. La hipertensión arterial y la diabetes *mellitus* tuvieron una menor prevalencia a la reportada por otros investigadores ^3^ ^-^ ^5^.

Entre los hallazgos clínicos y de laboratorio de los órganos evaluados por el SOFA, se encontró que la mediana de PaO_2_/FiO_2_ (103; 86 - 139) fue menor a la reportada por Ziehr *et al.* ^2^ y Søvik *et al.* ^3^. La necesidad de vasopresor al ingresar (34,3%) fue menor a la reportada por Ziehr *et al*. ^2^ y Ñamendys-Silva *et al*.^ (^ ^5^. La creatinina plasmática (0,7; 0,6 - 0,9 mg/dL) y la bilirrubina total (0,58; DE: 0,22 mg/dL) fueron menores, aunque el recuento plaquetario promedio (327,5; DE: 124,4 x10^3^/µL) fue mayor al descrito por Ñamendys-Silva *et al.* ^5^.

Los antibióticos al ingreso se usaron en el 34,3% de los pacientes y fue menor a lo reportado en Estados Unidos ^2^ y México ^5^. El 82,9% de los pacientes requirió VMI en posición prona y la indicación fue un PaO_2_/FiO_2_ ≤ 150 posintubación, la proporción de pacientes en VMI prona fue mayor a la descrita por otros autores ^2^ ^-^ ^5^. La programación de la VMI se hizo de acuerdo con el peso predicho del paciente ^1^, en caso de VMI en posición prona, la sesión duró 72 h.

La mortalidad (22,9%) fue menor a lo descrito en estudios semejantes ^4^ ^-^ ^6^ y mayor si se compara con estudios donde la mortalidad se valoró a los 30 días ^2^ ^,^ ^3^, los trabajos antes referidos se realizaron durante la primera ola y antes de la existencia de la vacuna para la COVID-19. Un paciente de nuestra serie había recibido la primera dosis de vacuna, tenía antecedente de trasplante de médula ósea por mieloma múltiple y falleció por la COVID-19. La probable explicación de la menor mortalidad en el presente estudio puede ser la menor edad de los pacientes, la experiencia adquirida en la primera ola, menor frecuencia de diabéticos y cardiópatas, no abuso de antibióticos al ingreso y la VMI de acuerdo con las características individuales. Ninguno de los pacientes que fallecieron en la UCI pudo ser retirado de VMI. La mediana de la estancia hospitalaria (25; 17-36 días) y de la UCI (17; 13-33 días) y fue semejante a la hallada por Søvik *et al.* ^3^ y mayor a lo descrito por Ñamendys-Silva *et al*. ^5^.

Nuestro estudio tiene las limitaciones propias de la metodología utilizada (observacional y retrospectivo), el tamaño de la muestra, la realización en un solo centro y la falta de exámenes de apoyo diagnóstico en algunos pacientes. 

En conclusión, nuestra población tuvo menor edad, recibieron VMI en posición prona con mayor frecuencia y la mortalidad fue menor, aunque con una mayor estancia hospitalaria y en UCI, cuando se comparó con otros reportes publicados.
